# The Development of a Clinical Research Model Complementing Medical Residency and the Assessment of Research Productivity

**DOI:** 10.7759/cureus.48684

**Published:** 2023-11-12

**Authors:** Satish C Nair, Khaled M Al-Dahmani

**Affiliations:** 1 Medical Research, Tawam Hospital, Al Ain, ARE; 2 Diabetes and Endocrinology, Tawam Hospital, Al Ain, ARE

**Keywords:** middle east, research productivity, research performance, research training, clinical trials, united arab emirates, gulf

## Abstract

Background and objective: Despite modern healthcare infrastructure, there is a paucity of information about the clinical research framework supporting healthcare in the United Arab Emirates (UAE). Therefore, this study aimed to assess research performance productivity, and the clinical research framework, complementing medical residency, at the nation’s hub for clinical research.

Methods: A cross-sectional retrospective review of records from the research database of the institution was conducted to assess productivity, and framework development, and data analyzed.

Results: The migration of global healthcare providers, and the pharmaceutical industry offices, the adoption of the International Conference on Harmonization (ICH) guidelines, and electronic medical records established a research culture. Following the development of the governance framework, a total of 1,328 research projects were submitted to the ethics committee until 2023. Approximately 63% of the total studies were of minimal risk, followed by industry-sponsored clinical trials (4.9%, 58/1,163), and prospective interventional studies (3.5%, 39/1,163). Almost half (48.3%, 28/58) of the total industry-sponsored clinical trials were phase II and phase III. The number of peer-reviewed indexed publications, a measure of research productivity, indicated that the periods between 2011 and 2015, 2016 and 2020, and 2021 and 2023 witnessed a 3.8-, 9.3-, and 7.9-fold increase compared to the baseline period (1995-2005). The implementation of the Focus on International Research Strategy, Teaching, Evaluation, and Mentoring (FIRSTEM) strategy, to accommodate mandatory research activity requirements for residents by the physician licensing boards, observed substantial increases in output. The number of international peer-reviewed indexed publications/resident projects doubled from 10.8% (2010-2015) to 24% (2016-2020) and reached 40.1% in 2023.

Conclusion: This is the first research governance model established in the UAE, a country with an increasing prevalence of diabetes, and cardiovascular and genetic diseases. The model indicates that the medical trainees differentiate the best research evidence in making decisions about the clinical care of patients. The study outcomes may potentially be useful for other countries in developing a clinical research framework.

## Introduction

Trade globalization, tourism, and people migration have enhanced the potential for the spread of a spectrum of diseases, thus necessitating the need to conduct clinical research in international settings [1]. Clinical research, beyond clinical understanding and disease prevention, also provides the foundation to track disease incidence in the population and predict cause-and-effect relationships [2]. Strategic investments in building research capacity have been associated with improvement in the standards of living and quality of life and long-term sustainable economic advancement [3]. The development of a clinical research framework in an international setting is not without obstacles. Inefficient healthcare systems to support clinical research, inadequate research infrastructure and investments, lack of institutional ethics committees (IECs) and regulatory oversight, untrained workforce, and poor data protection systems pose insurmountable challenges [4].

The United Arab Emirates (UAE) is a young nation, established in 1971 as a federation of seven emirates: Abu Dhabi, Dubai, Ajman, Umm Al Quwain, Sharjah, Fujairah, and Ras Al Khaimah. The UAE transformed from a traditional to a state-of-the-art healthcare provider since the year 2005, following the government directive [5]. The World Health Organization estimates that approximately 19% of the UAE population is diabetic and that 38% of the annual mortality rate in the country can be attributed to diabetes, cancer, and cardiovascular diseases. Almost 60% of the population is overweight, and there is a higher prevalence of rare genetic diseases from consanguinity (WHO) [5,6]. Traditionally, the UAE has chosen to be a consumer of clinical research rather than a producer [7]. Despite the government’s vision to transform the UAE into a knowledge-based economy by 2030, very limited information about clinical research, an integral component of the healthcare infrastructure, is a challenge [7]. The development of a clinical research framework in the UAE, in addition to the global challenges, also includes local barriers such as the following: (a) absence of a research culture, (b) patient apathy toward research participation, (c) limited community awareness for research, and (d) perception of some academic physicians that clinical research is not truly an intellectual endeavor [8]. Given that limited information about the clinical research framework supporting healthcare in the United Arab Emirates is known, this study aimed to assess the clinical research framework at Tawam Hospital, the nation’s hub for clinical research, and to assess research productivity. It is anticipated that the study outcomes will potentially serve as a road map for other countries in the international setting in the development of a comprehensive clinical research model.

## Materials and methods

Study setting

The study was conducted at a 470-bed, Joint Commission- and Accreditation Council for Graduate Medical Education International (ACGME-I)-accredited tertiary care public hospital in the eastern region of the emirate of Abu Dhabi, United Arab Emirates. The hospital caters to the secondary and tertiary healthcare needs of almost one million people in the city of Al Ain, and others spread across the suburbs of Al Ain city, and the country in general. Established in 1979, the hospital provides pediatric to adult care, across almost all specialties.

Study design and data collection

Historical records relating to research regulatory notifications, guidelines, decrees, and national laws were examined to assess the research ecosystem, ethics framework, and regulatory landscape prior to the year 2005. The research database was accessed following research ethics committee approval. The research database is continuously updated for study-related information, patient participant information, financials, adverse events, good standing and professional licenses of physicians, indexed publications, investigator expertise, start and stop dates, amendments to protocol, and extensions. Data was abstracted retrospectively and included the types and number of studies approved by the research ethics committee, the reasons for rejection by the ethics committee, the number of medical resident research projects and publications, the total number of peer-reviewed indexed publications, and research training records. Healthcare institutions within the Abu Dhabi Health Services company system, the operator of 13 public hospitals and several primary care clinics in the UAE, are mandated to meet annual research productivity criteria. A total of five research productivity performance criteria were established to assess clinical research productivity and performance in each of the healthcare facilities (internal communication): (a) the number of research publications in peer-reviewed indexed journals, (b) the number of research publications by the major specialties in peer-reviewed indexed journals, (c) the number of publications by medical residents as the percentage of total resident projects, (d) the total number of research training programs conducted, and (e) accomplishment of successful accreditation/regulatory review compliance. The research productivity performance criterion target for Tawam Hospital (revised annually) was developed based on the number of (a) specialties, (b) certified research professionals, and (c) hospital beds. Data was abstracted by three independent researchers unaware of the study objectives to eliminate bias. The quality of data abstracted by the researchers was subjected to an inter-rater reliability test, Cohen’s kappa [9]. A Cohen’s kappa score of >0.80 reflected agreement between the data collectors and was recorded in password-protected Excel sheets (Microsoft Corporation, Redmond, WA). The period of data collection was between January 5, 2023, and *June 5, 2023. Various data were collected for the years between 1995 and June 2023, spread over 28 years, and were further subjected to descriptive data analysis, and results are presented as mean ± standard deviation (SD).

Ethics approval

The methods of the study were carried out in accordance with the International Conference on Harmonization (ICH) and Good Clinical Practice guidelines, and the local and national guidelines from the Department of Health, Abu Dhabi, UAE. The study obtained research ethics approval from the Tawam Hospital Human Research Ethics Committee, a centralized research ethics committee, vide approval number 915/2022. At every stage of the data collection, data confidentiality was adhered to, to prevent misuse of institutional data.

## Results

Culture of research

The review of the historical regulatory records from Tawam Hospital indicated that clinical research was not formally an organized activity for the health facilities before the year 2005. The Tawam Hospital, established in the year 1979, provided care in almost all areas of medicine, ranging from family medicine to maxillofacial surgery. Tawam Hospital served as the largest regional referral center for oncology, surgical, medical, radiation oncology, and palliative care. Regardless of the fact that Tawam Hospital, a teaching hospital, trained medical trainees from the UAE University College of Medicine and Health Sciences, structured clinical research was lacking until 2006. Traditionally, the focus of academic researchers was basic sciences research [10]. The healthcare transformation initiated by the UAE government in the year 2005 also witnessed the migration of a large number of global healthcare providers to the UAE. Global healthcare providers such as the Johns Hopkins Medicine, Cleveland Clinic, and others, competent in the academic triad, namely, patient care, education, and research, fueled the culture of research to pursue excellence in healthcare [11]. Western-trained physician migration from various countries, the transformation of the UAE healthcare system, particularly the implementation of electronic medical records, and the willingness of global pharmaceutical industries to conduct studies at Tawam Hospital also contributed to the culture of research after 2006 [11].

Ethics and regulations

The modernization of the healthcare system, recruitment of a Western-trained research director, and establishment of a clinical research department at Tawam Hospital initiated the development of a research strategy and operations in the emirate of Abu Dhabi, in particular in Tawam Hospital [12]. Subsequently, the Ministry of Health in the UAE adopted the International Conference on Harmonization and Good Clinical Practice guidelines, setting the stage for the development and implementation of national research [13]. The Institutional Ethics Committee at Tawam Hospital was constituted according to regulatory guidelines and consisted of various stakeholders: physicians, nurses, and a layperson. The license to conduct responsible human clinical research was obtained from the Department of Health, Abu Dhabi [11]. The number of research proposal submissions for ethical consideration increased from 7% of total submissions in 2008-2011 to 36.1% in 2020-2023 (Table 1). A total of 1,328 research proposals were submitted to the Tawam Hospital IEC from June 2008 to June 30, 2023, for ethics review. The average turnaround time required for ethics review and decision, starting from document compliance check, was 20 days (19.9 ± 3.2) for minimal risk studies and 44 days (44.1 ± 6.8) for industry-sponsored clinical trials and interventional research studies. The rejection rate following the ethics review was approximately 12.5% (1,163/1,328). Inadequate adoption of human subject protection mechanisms and lack of a robust research design were the main reasons for unfavorable decisions by the IEC.

**Table 1:** Increasing number of research proposal submissions to the human research ethics committee for favorable ethical consideration (N = 1,328) A total of 1,328 clinical research projects have been submitted to the Institutional Research Ethics Committee for ethical consideration since the inception of the clinical research model in the year 2008. Of the total research proposal submissions (1,328), 7% were during the period between 2008 and 2010, which increased to 12%, 22.1%, 22.7%, and 36.1% of the total submissions during the periods between 2011 and 2013, 2014 and 2016, 2017 and 2019, and 2020 and 2023, respectively. *Data was collected until June 2023.

Period	Total Clinical Research Proposal Submissions for Ethical Decision	Percentage of Total
2008-2010	93	7
2011-2013	159	12
2014-2016	294	22.1
2017-2019	302	22.7
2020-2023*	480	36.1

Unified research gateway

**Figure 1:**
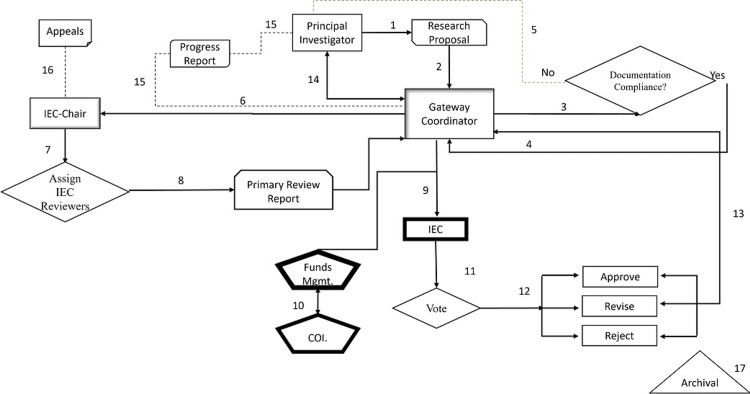
Clinical research gateway Steps 1-17 represent the submission, screening, selection, ethics review, decision-making, and archiving of research proposals through the clinical research gateway. IEC: institutional ethics committee, Funds Mgmt: funds management committee, COI: conflicts of interest committee, Archival: archival system

A unified research gateway is critical to provide a secure data platform with analytical and orchestration tools to exchange research materials and updates between the stakeholders and regulators. The Tawam Research Gateway was designed and developed at Tawam Hospital in 2009 (Figure 1).

**Figure 2:**
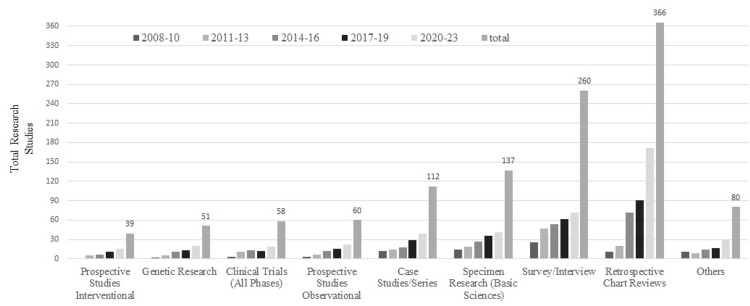
Various types of clinical research studies approved by the institutional human research ethics committee (N = 1,163) from 2008 to June 2023 A total of 1,163 research studies received favorable ethics decisions until June 2023. The rejection rate was approximately 12% (1,163/1,328). Of the total studies, 60% (738/1,163, 63.7%) were of minimal risk, such as retrospective observational studies, surveys and interviews, and case reports. Industry-sponsored clinical trials contributed to 4.9% (58/1,163), prospective interventional studies 3.5% (39/1,163), and prospective observational studies 5.1% (60/1,163) of the total number of research projects. Research using patient specimens, and genetic research, tissue, and cells contributed to 4.4% (51/1,163) and 11.8% (137/1,163) of the total projects, respectively.

The gateway consisted of eight interdependent modules: (a) proposal submission, (b) ethics review, (c) appeals, (d) proposal tracking, (e) research funds management, (f) conflicts of interest in research (CORE), (g) progress reports, and (h) archival system [14]. In brief, the research proposals are submitted by the principal investigators in a prescribed application form, developed by the Department of Health, Abu Dhabi. The research proposals are screened for regulatory documentation compliance by the gateway coordinator, followed by anonymized reviews by two subject expert reviewers, nominated by the chair of the IEC. The review report from the reviewers is then shared with all the members of the IEC, the funds management, and the conflicts of interest group and voted toward an ethical decision. Votes are recorded electronically, reflecting approval, rejection, or revision requested, within 72 hours of receiving the review reports. The number of votes is tallied by the coordinator and shared with the IEC chair and, subsequently, the IEC (Figure 1). The majority votes qualify for a favorable ethical decision. The investigators are mandated to provide an annual research progress report and seek ethics approval for major protocol amendments and study period extensions. An upward trend in the number and type of studies was noted starting from 2008; a total of 1,163 research studies received favorable ethics decisions until 2023 (Figure 2). Over 60% (738/1,163, 63.7%) of the total studies were of minimal risk, such as retrospective observational studies, surveys and interviews, and case reports. Industry-sponsored clinical trials contributed to 4.9% (58/1,163), prospective interventional studies 3.5% (39/1,163), and prospective observational studies 5.1% (60/1,163) of the total number of research projects (Figure 2). Approximately half (48.3%, 28/58) of the total industry-sponsored clinical trials were phase II and phase III (data not shown). Research using patient specimens, and genetic research, tissue, and cells contributed to 4.4% (51/1,163) and 11.8% (137/1,163) of the total projects, respectively. 

Collaboration and partnerships

The higher prevalence of genetic diseases, wider spectrum of the disease pool, and inadequate prenatal screening in the UAE justify the need for a robust clinical research system. Interestingly, around the year 2005, the exorbitant cost of conducting clinical trials in North America prompted the major pharmaceutical companies to conduct large trials overseas [5,15]. It was estimated that the total trial costs including patient follow-up over lengthy periods were economical, aided by outstanding health infrastructure, in the UAE [5,16]. The research division and the hospital administration capitalized on this opportunity to provide a platform for global pharmaceutical companies to conduct studies at Tawam Hospital, especially in the areas of oncology, medicine, and pediatrics. The first global pediatric clinical trial, phase II, industry-sponsored and initiated in 2009, was completed, following the United States Food and Drug registration and approval in 2015 [17,18]. Tawam Hospital, with no citations, was recognized as the best clinical trial site outside the USA [18]. A total of 49 clinical trials have been completed between the period of 2009 and 2022, the majority being in the area of oncology. Several observational and interventional clinical trials are currently ongoing (data not shown). The pharmaceutical industry in 2017 recognized Tawam Hospital as the “Most Favored Trial Site,” given the ease of conducting a study in a professional setting. International collaborations with several hospitals and medical centers in the United States, United Kingdom, Germany, Netherlands, Jordan, and other countries in the Middle East were established in 2009, evidenced by the joint peer-reviewed indexed publications [19,20].

Research productivity performance

Publications

**Figure 3:**
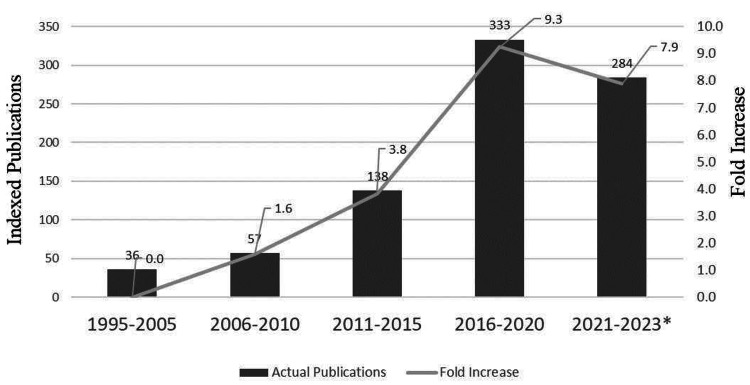
Total number of peer-reviewed indexed publications and the fold increase in publications compared to the baseline period (1995-2005) (N = 848) Research productivity performance was measured by the number of peer-reviewed indexed publications. The publications for the period 1995-2005 served as the baseline. Compared to the baseline, a 1.6-fold increase in the number of indexed publications was observed for the period 2005-2010, following the inception of the clinical research model in 2008. The periods between 2011 and 2015, 2016 and 2020, and 2021 and 2023 witnessed a 3.8-, 9.3-, and 7.9-fold increase in the number of peer-reviewed indexed publications, respectively. *Data was collected until June 2023.

Research productivity evidenced by the number of peer-reviewed indexed publications was limited to 36 publications for the 10 years between 1995 and 2005. A slow start to the increase in publications was noted following the inception of the clinical research governance model in the year 2008 at Tawam Hospital. Approximately a 1.6-fold (57/36) increase in the number of indexed publications was observed for the period 2005-2010 (Figure 3). The periods between 2011 and 2015, 2016 and 2020, and 2021 and 2023 witnessed a 3.8-, 9.3-, and 7.9-fold increase in the number of peer-reviewed indexed publications, respectively, compared to the baseline period (1995-2005) (Figure 3). Internal medicine contributed 50% (427/848) of the total indexed publications; nephrology, neurology, dermatology, endocrinology, and rheumatology, in particular, were the top five contributors. Nearly 12% of the publications were from oncology (medical, radiation, and palliative care). Pediatrics including neonatology (10%), emergency medicine (9%), and laboratory medicine (8%) were the other major contributors to the publications.

Medical Residency Research

**Figure 4:**
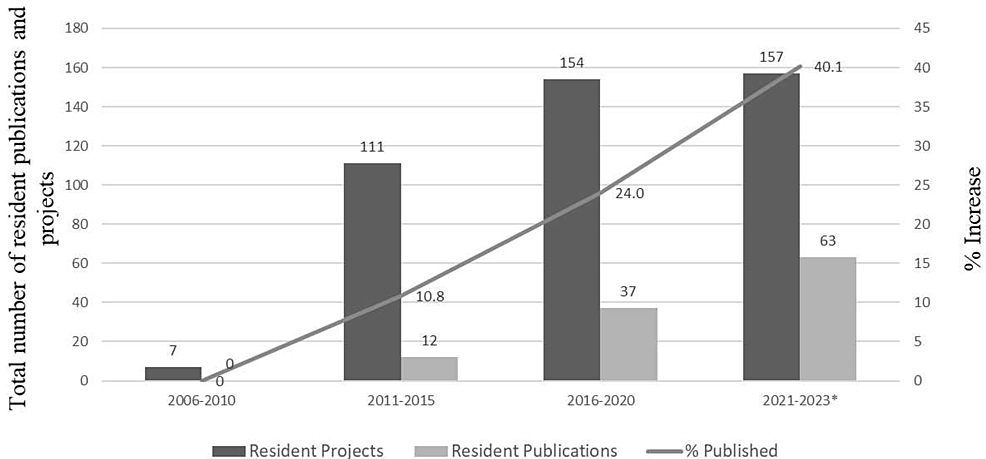
Number of international peer-reviewed indexed publications and the publication rate (number of international peer-reviewed indexed publications/resident projects) by the medical residents The periods between 2016 and 2020, and 2021 and 23 witnessed substantial increases in medical trainee projects and publications. The number of international peer-reviewed indexed publications/resident projects (publication rate) by medical residents doubled from 10.8% (2010-2015) to 24% (2016-2020) and reached 40.1% in 2023, following the implementation of the FIRSTEM. *Data was collected until June 2023. FIRSTEM: Focus on International Research Strategy, Teaching, Evaluation, and Mentoring

Tawam Hospital developed and implemented the Focus on International Research Strategy and Teaching (FIRST), a hospital-wide approach to research strategy and training in 2012 [12]. The FIRST program provided a common platform for medical trainees to engage in Good Clinical Practice training and certification. Three years (2012-2015) following the implementation of the FIRST program, the number of medical resident projects increased to 111 and resulted in 12 indexed publications, and the publication rate (number of resident publications/numbers of resident projects) increased by 10.8%, compared to zero medical trainee publications during the preceding period (2005-2010) (Figure 4). The medical residency research program was re-evaluated in 2017, five years after the FIRST implementation, to accommodate the mandatory research activity requirement for residents by the various physician licensing boards and the global accreditation bodies [21]. The FIRST program was refurbished to Focus on International Research Strategy, Teaching, Evaluation, and Mentoring (FIRSTEM), incorporating five additional modules, namely, resident-mentor matching, resident participation in research ethics committees, resident research blocks, statistical analysis exposure, and continuous in-house research methodology training [21]. Following FIRSTEM, the periods between 2016 and 2020, and 2021 and 2023, Tawam Hospital witnessed substantial increases in medical trainee projects and publications. The number of international peer-reviewed indexed publications/resident projects (publication rate) by medical residents doubled from 10.8% (2010-2015) to 24% (2016-2020) and reached 40.1% in 2023 (Figure 4).

Research Training

The Clinical Research Excellence and Development of Innovation and Technology (CREDIT) training workshop program was designed and developed by the clinical research department at Tawam Hospital [22]. The program, initially developed for medical trainees, was extended for all medical professionals, and clinical pharmacists conducting research, across the UAE hospitals and universities. The CREDIT training workshop included six modules, namely, (a) ideation and literature search, (b) research question development, (c) designing studies, (d) research ethics and regulations, (e) statistical analysis, and (f) scientific writing and critiquing, spread over six sessions and 48 contact hours, followed by certification [22]. A total of 1,893 (2016-2020) participants and 971 (2021-2023*) completed the training program (data not shown).

Accreditations and Regulatory Reviews

The clinical research enterprise of Tawam Hospital has been reviewed for the human subject research program standards every three years, starting from the year 2009. Components of the research standards review involve ethics, human subject protection, regulatory compliance, communication, leadership, transition of care, and conflicts of interest. The Joint Commission reviews in 2009, 2012, 2015, 2018, and 2021 were completed without the requirement for improvement citation by the assessors. The medical residency programs in Tawam Hospital achieved accreditation through the Accreditation Council for Graduate Medical Education International (ACGME-I) beginning in 2012. The ACGME-I mandates residents and fellows to engage in scholarly activities including, but not limited to, conducting research, writing scientific manuscripts, presenting findings at conferences, and critiquing scientific publications.

## Discussion

Less than 0.1% of the global clinical trials have been reported from the Middle East region [5]. Historically, clinical research productivity among the Gulf Cooperation Countries in the Middle East, except for Saudi Arabia, has been exceedingly low [23,24]. The paucity of structured information related to clinical research models and inadequate sustainability, two key implementation outcomes, have contributed to the low research productivity in the other Gulf Cooperation Countries [5,7]. The UAE government healthcare initiatives, the migration of global healthcare providers to the UAE, competition among the providers, and the implementation of electronic medical records served as the cornerstones in the development of a clinical research program at Tawam Hospital. The adoption of the International Conference on Harmonization and Good Clinical Practice guidelines, national research regulations by the Department of Health, Abu Dhabi, licensing of research facilities, and the establishment of investigator(s) criteria to conduct research paved the path to organizing a culture of research [5,7,13]. This was evidenced by the exponential growth in research proposal submissions. The number of research proposal submissions for ethical consideration increased from 7% of total submissions in 2008-2011 to 36.1% in 2020-2023 (Table 1). A total of 1,328 research proposals were submitted to the IEC during this period. Although the vast majority (63.7%) of the research studies were of minimal risk, the industry-sponsored clinical trials, prospective studies, and genetic studies each contributed to 5% or less of the total (Figure 2).

An efficient healthcare system should support research infrastructure. The Tawam Research Gateway was aimed at connecting two broad functions of the clinical research system, interaction with the researchers and the expertise of the regulators, and their contexts to address problems, from the experiences of a rapidly changing healthcare sector [25]. Despite the growing concerns that the institutional ethics committees, globally, impose burdensome delays in the review and approval of research studies, the average turnaround time from submission to a decision for the Tawam Hospital IEC was 19.2 ± 3.2 days (mean ± SD) for minimal risk studies and 43.7 ± 6.8 days for industry-sponsored clinical trials and interventional research studies. The review times for the Tawam Hospital IEC were within the 60-day target recommended by global expert panels [26]. Clinical research is complex and knowledge-intensive, and patients often assume that their care providers use the best available knowledge on diagnosis and treatment. University research attempts for knowledge production, whereas hospitals seek a faster, systematic use of knowledge hinging on knowledge transfer and translation [27]. The Tawam Hospital research strategy was focused on developing partnerships and collaborations between the hospitals and academia to enable and enhance the use of research and increase the amount of research relevant to patients. Research using patient specimens for oncology and genetic diseases were the highlight of academic collaboration [28,29]. Additionally, partnerships and collaborations with the healthcare system are the sustainable development goals for the pharmaceutical industry [30]. The industry collaborations enabled clinical trials in the areas of imaging, breast and lung oncology, diabetes, and cardiovascular, rheumatic, pediatric, and genetic diseases. Research, education, and patient care are important cornerstones of academic medical centers and their teaching programs. Quantification and evaluation of clinical research productivity are essential measures for the sustainability of a clinical research model [31].

Several measures have been globally adopted to assess research productivity, including the quantification of indexed scientific publications [32]. Compared to the baseline period (1995-2005) (Figure 3), the period between 2011 and 2023 recorded approximately an overall 21-fold increase in the number of indexed publications, the major contributor being internal medicine. Teaching hospitals struggle with ways to effectively support quality research programs, particularly in countries with developing medical education systems, given the absence of uniformity in the backgrounds and experience of the faculty and varying training programs [12]. Interestingly, the Focus on International Research Strategy and Teaching (FIRST) program enabled the organization of a basic resident research program at Tawam Hospital. The refurbished program, Focus on International Research Strategy, Teaching, Evaluation, and Mentoring (FIRSTEM), aided in significantly enhancing the resident research publication rate by approximately 40% (Figure 4). The Clinical Research Excellence and Development of Innovation and Technology (CREDIT) workshop training program provided the major thrust to exceed the desired research outcomes, such as quality publications, higher medical resident pass rate for the professional licensing examinations, international recognition, and the quality conduct of clinical trials. The Joint Commission and other accreditations are often seen as a platform for hospitals to maintain a higher level of quality and governance through timely problem-finding, improved communication, effective departmental collaboration, uniformity of standards, and heightened branding. The outcomes from several international accreditations and regulatory reviews and audits reflect the transparency, utility, and robustness of the clinical research governance model at Tawam Hospital. The Association of Clinical Research Professionals, USA, in 2013, and recently the UAE Ministries of Health and Prevention (2023) have recognized and awarded the clinical research model at Tawam Hospital [33,34].

Limitations and strengths

Our study has several strengths: (a) the clinical research model and governance system is the first in the UAE and perhaps among the Gulf Cooperation Countries, (b) the research productivity outcomes indicate that the clinical research model complements medical residency and enables the trainees to differentiate best research evidence in making decisions about clinical care, and (c) the sustainability of the model for over 15 years and the outcomes of the study are anticipated to generate several health policy reforms toward evidenced-based care. The fact that this is a single-center study and that the impact of the research by the medical trainees to improve patient care remains unknown are the limitations of the study. The model was established at a tertiary care, Joint Commission-accredited, public hospital and the nation’s largest hub for clinical trials and clinical research, and thus may enable generalization and partially overcome the single-center limitation.

## Conclusions

The establishment of a sustainable clinical research model in international settings is not without challenges. The clinical research model and governance system at Tawam Hospital is perhaps the first one in the country and the region. The clinical research model has generated a robust research project submission and review portal for the end users, managing both financial and non-financial conflicts of interest, with a built-in research methodology training and mentoring program for medical professionals and trainees in particular. The research productivity performance portal has enabled the assessment and evaluation of research productivity, such as the number of research project submissions, rejection rates following ethics review, the number and type of clinical trials, the number of indexed publications, international regulatory review compliance, and research training and mentoring. The establishment of a sustainable clinical research model and governance system is critical for the UAE given the fact that 19% of the UAE population is diabetic and that diabetes, cancer, and cardiovascular diseases account for 38% of the total annual mortality rate. A robust clinical research program is anticipated to reform health policies and inform local guidelines for evidence-based care management.

## References

[1] Klímová B, Kuča K (2020). Medical tourism: its research and implications for public health. Cent Eur J Public Health.

[2] Politi MC, Lewis CL, Frosch DL (2013). Supporting shared decisions when clinical evidence is low. Med Care Res Rev.

[3] Dako-Gyeke P, Asampong E, Afari E (2020). Capacity building for implementation research: a methodology for advancing health research and practice. Health Res Policy Syst.

[4] Olopade CO, Olugbile S, Olopade OI (2012). Issues and challenges for clinical research in international settings. Principles and Practice of Clinical Research.

[5] Nair SC, Ibrahim H, Celentano DD (2013). Clinical trials in the Middle East and North Africa (MENA) region: grandstanding or grandeur?. Contemp Clin Trials.

[6] Sulaiman N, Elbadawi S, Hussein A (2017). Prevalence of overweight and obesity in United Arab Emirates Expatriates: the UAE National Diabetes and Lifestyle Study. Diabetol Metab Syndr.

[7] Ibrahim H, Kamour AM, Harhara T, Gaba WH, Nair SC (2020). Covid-19 pandemic research opportunity: is the Middle East & North Africa (MENA) missing out?. Contemp Clin Trials.

[8] Al-Shami KM, Ahmed WS, Alzoubi KH (2022). Motivators and barriers towards clinical research participation: a population-based survey from an Arab MENA country. PLoS One.

[9] McHugh ML (2012). Interrater reliability: the kappa statistic. Biochem Med (Zagreb).

[10] Alahmad G, Al-Jumah M, Dierickx K (2012). Review of national research ethics regulations and guidelines in Middle Eastern Arab countries. BMC Med Ethics.

[11] Nair SC, Ibrahim H (2015). Assessing subject privacy and data confidentiality in an emerging region for clinical trials: United Arab Emirates. Account Res.

[12] Ibrahim H, Nair SC (2014). Focus on international research strategy and teaching: the FIRST programme. Perspect Med Educ.

[13] Ibrahim Ibrahim, K. K., Eada Eada, E. A., and Younis, N. (2006 (2023). United Arab Emirates Ministry Of Health Drug Control Department (DCD): Guidance for conducting clinical trials based on drugs/medical products & good clinical practice. on drugs/medical products and Good Clinical Practice.

[14] Nair SC, AlGhafli S, AlJaberi A (2018). Developing a clinical trial governance framework for pharmaceutical industry-funded clinical trials. Account Res.

[15] Glickman SW, McHutchison JG, Peterson ED, Cairns CB, Harrington RA, Califf RM, Schulman KA (2009). Ethical and scientific implications of the globalization of clinical research. N Engl J Med.

[16] (2023). IQVIA: Unearthing the potential of the clinical trial market in Middle East, Turkey and Africa (META) region. https://www.iqvia.com/locations/middle-east-and-africa/library/white-papers/unearthing-the-potential-of-clinical-trial-market-in-mena.

[17] Reis FS, Lazaretti-Castro M (2023). Hypophosphatasia: from birth to adulthood. Arch Endocrinol Metab.

[18] Whyte MP, Simmons JH, Moseley S (2019). Asfotase alfa for infants and young children with hypophosphatasia: 7 year outcomes of a single-arm, open-label, phase 2 extension trial. Lancet Diabetes Endocrinol.

[19] (2023). SEHA, MBZUAI to collaborate on clinical research. SEHA, MBZUAI to collaborate on.

[20] (2023). Johns Hopkins and SEHA to collaborate on enhancing patient safety. https://www.hopkinsmedicine.org/news/media/releases/johns_hopkins_and_seha_to_collaborate_on_enhancing_patient_safety.

[21] Nair SC, Ibrahim H, Almarzoqi F, Alkhemeiri A, Sreedharan J (2019). Addressing research barriers and facilitators in medical residency. J Family Med Prim Care.

[22] Nair SC, Satish KP, Ibrahim H (2021). Programme to improve medical resident research in a developing health care system. Med Educ.

[23] Al-Hajri A, Al-Khabori M, Rasool W (2022). Productivity of clinical trials conducted in the Gulf Cooperative Council region. Sultan Qaboos Univ Med J.

[24] Al-Rawashdeh N, Damsees R, Al-Jeraisy M, Al Qasim E, Deeb AM (2019). Knowledge of and attitudes toward clinical trials in Saudi Arabia: a cross-sectional study. BMJ Open.

[25] Montori VM, Hargraves I, McNellis RJ (2019). The care and learn model: a practice and research model for improving healthcare quality and outcomes. J Gen Intern Med.

[26] Hall DE, Hanusa BH, Stone RA, Ling BS, Arnold RM (2015). Time required for institutional review board review at one Veterans Affairs medical center. JAMA Surg.

[27] Kruse P, Kummer C, Jannack A (2015). Empowering knowledge transfer in healthcare: a framework of knowledge transfer methods. Challenges and opportunities in health care management.

[28] Hassan IB, Kristensen J, Alizadeh H, Bernsen R (2013). Outcome of patients with acute lymphoblastic leukemia (ALL) following induction therapy with a modified (pulsed dexamethasone rather than continuous prednisone) UKALL XII/ECOG E2993 protocol at Tawam Hospital, United Arab Emirates (UAE). Med Oncol.

[29] Al-Shamsi A, Hertecant JL, Souid AK, Al-Jasmi FA (2016). Whole exome sequencing diagnosis of inborn errors of metabolism and other disorders in United Arab Emirates. Orphanet J Rare Dis.

[30] Olk P, West J (2019). The relationship of industry structure to open innovation: cooperative value creation in pharmaceutical consortia. RD Manag.

[31] Wildgaard L, Schneider JW, Larsen B (2014). A review of the characteristics of 108 author-level bibliometric indicators. Scientometrics.

[32] Pal J, Sarkar S (2020). Evaluation of institutional research productivity. DESIDOC J Libr Inf.

[33] (2023). ACRP appoints chair of GCC. https://scrip.citeline.com/SC096562/ACRP-appoints-chair-of-GCC.

[34] (2023). MoHAP unveils "Landscape of Health Research Report in the UAE 2017-2022". https://mohap.gov.ae/en/media-center/news/1/8/2023/mohap-unveils-landscape-of-health-research-report-in-the-uae-2017-2022.

