# Correction: The Development of a Clinical Research Model Complementing Medical Residency and the Assessment of Research Productivity

**DOI:** 10.7759/cureus.c154

**Published:** 2024-01-19

**Authors:** Satish C Nair, Khaled M Al-Dahmani

**Affiliations:** 1 Medical Research, Tawam Hospital, Al Ain, ARE; 2 Diabetes and Endocrinology, Tawam Hospital, Al Ain, ARE

This article has been corrected at the request of the authors to change the affiliations from Johns Hopkins Medicine International, Tawam Hospital, Al Ain, ARE to Tawam Hospital, Al Ain, ARE.

The authors deeply regret that these errors were not identified and addressed prior to publication.

 

